# Correspondence: *FKBP5* blockade may provide a new horizon for the treatment of stress‐associated disorders: An in silico study

**DOI:** 10.1002/epi4.12813

**Published:** 2023-09-28

**Authors:** Xiang Shang, Xiaoming Sun, Shuman Pan

**Affiliations:** ^1^ Department of Pharmacy Suzhou Guangji Hospital, The Affiliated Guangji Hospital of Soochow University Suzhou China

Dear Editor,

We read with great interest the article by Asadi‐Pooya et al.^1^ The study conducted by the researchers to investigate the potential use of FKBP5 inhibitors for the treatment of stress‐related disorders, such as functional seizures (psychogenic nonepileptic seizures/attacks) by using bioinformatics tools and databases. The study identified 28 FKBP5 inhibitors (such as clozapine, sertraline, etc.) that could be used for clinical trials in patients with stress‐related disorders. However, regarding this study, we think that there are some aspects worth paying attention.

First, it is well known that clozapine could reduce the seizure threshold and provoke seizures.^2^, ^3^

Because of the highest risk of seizures, treatment with clozapine is avoided or done with caution in patients with psychosis and seizures.^4^ In addition, as one of the most effective antipsychotic drugs, clozapine is strictly restricted from use because it may lead to rare adverse reactions such as agranulocytosis.^5^ Although functional seizures (psychogenic nonepileptic seizures/attacks) are not associated with electrophysiological epileptic changes, it is necessary for us to clarify these facts and cautiously expand the use of clozapine.

Second, sertraline, as an antidepressant, is commonly prescribed to treat psychiatric disorders such as depression, panic disorder, generalized anxiety disorder (GAD), social anxiety disorder, and obsessive‐compulsive disorder (OCD).^6^ The authors highlighted that sertraline was an acceptable candidate drug for the treatment of functional seizures (psychogenic nonepileptic seizures/attacks). However, there are few studies to explore the relationship between sertraline and FKBP5 so far. A study analyzed the association between the *FKBP5* gene and sertraline in patients with major depressive disorder (MDD). However, no significant effect of sertraline on *FKBP5* gene expression was observed before starting treatment with antidepressants and after 8 weeks of taking medication.^7^ Recently, a study investigated the relationship between *FKBP5* single‐nucleotide polymorphisms (SNPs) and functional seizures (psychogenic nonepileptic seizures/attacks).^8^ The study found that there were no significant differences in genotype and allelic frequencies between the functional seizures (psychogenic nonepileptic seizures/attacks) and MDD groups. So far, the relationship between sertraline, *FKBP5* polymorphisms, and functional seizures (psychogenic nonepileptic seizures/attacks) is still unclear. Therefore, there are still great challenges for sertraline in the treatment of functional seizures (psychogenic nonepileptic seizures/attacks).

Additionally, clinical features are not specific enough to definitively differentiate epileptic seizures from functional seizures (psychogenic nonepileptic seizures/attacks). Psychotherapeutic interventions are the primary approach used to treat functional seizures (psychogenic nonepileptic seizures/attacks). Treatment modalities of functional seizures (psychogenic nonepileptic seizures/attacks) mainly contain cognitive behavioral therapy (CBT), CBT‐informed psychotherapy, psychodynamic therapy, and group therapies.^9^ Although drug therapy (especially antidepressants) has been explored for the treatment of functional seizures (psychogenic nonepileptic seizures/attacks) with psychiatric comorbidities, the therapeutic effect was not ideal.^10^ Furthermore, the identified drugs, especially antipsychotics and antidepressants, usually need to be taken for long‐time administration and adverse drug reactions are an important factor affecting treatment.

In general, we greatly appreciate the authors' exploratory work. The identified drugs could potentially be verified in clinical trials in patients with functional seizures (psychogenic nonepileptic seizures/attacks). Considering the lack of sufficient evidence‐based evidence, more studies are needed to investigate the relationship between FKBP5 and the identified drugs, which would be a huge challenge.

## FUNDING INFORMATION

This work was supported by grants from SuZhou Municipal Health Commission (Qngg2022029).

## CONFLICT OF INTEREST STATEMENT

The authors have no relevant conflict of interest to disclose. We confirm that we have read the Journal's position on issues involved in ethical publication and affirm that this report is consistent with those guidelines.
